# Population genetic structure in a social landscape: barley in a traditional Ethiopian agricultural system

**DOI:** 10.1111/eva.12091

**Published:** 2013-08-12

**Authors:** Leah H Samberg, Lila Fishman, Fred W Allendorf

**Affiliations:** 1 University of CaliforniaSanta Cruz, CA, USA; 2 University of MontanaMissoula, MT, USA

**Keywords:** agriculture, conservation genetics, crop genetic resources, ethiopia, farmer seed systems, landraces

## Abstract

Conservation strategies are increasingly driven by our understanding of the processes and patterns of gene flow across complex landscapes. The expansion of population genetic approaches into traditional agricultural systems requires understanding how social factors contribute to that landscape, and thus to gene flow. This study incorporates extensive farmer interviews and population genetic analysis of barley landraces (Hordeum vulgare) to build a holistic picture of farmer-mediated geneflow in an ancient, traditional agricultural system in the highlands of Ethiopia. We analyze barley samples at 14 microsatellite loci across sites at varying elevations and locations across a contiguous mountain range, and across farmer-identified barley types and management strategies. Genetic structure is analyzed using population-based and individual-based methods, including measures of population differentiation and genetic distance, multivariate Principal Coordinate Analysis, and Bayesian assignment tests. Phenotypic analysis links genetic patterns to traits identified by farmers. We find that differential farmer management strategies lead to markedly different patterns of population structure across elevation classes and barley types. The extent to which farmer seed management appears as a stronger determinant of spatial structure than the physical landscape highlights the need for incorporation of social, landscape, and genetic data for the design of conservation strategies in human-influenced landscapes.

## Introduction

In all kinds of landscapes throughout the world, the extent to which populations are structured across space and time is mediated by pathways of connectivity and physical, behavioral, and temporal barriers to dispersal. The past decade has seen the development of a suite of tools with which to understand the processes and patterns of gene flow across complex landscapes, and the ways in which landscape features affect the structure of populations (Manel et al. 2003). These tools are especially relevant in an era of rapid environmental and land-use change, as population fragmentation or loss of diversity may reduce the potential for populations to adapt to changing conditions (Grivet et al. 2008).

A population genetics approach to conservation has been expanded to include research into traditional agricultural systems, where decisions by farmers regarding how and where to access diversity can determine patterns of geneflow, as well as isolation, drift, and local adaptation (Parzies et al. 2004; Yahiaoui et al. 2008; Berthouly et al. 2009). This study combines farmer interviews with conservation genetic methods and phenotypic analysis to investigate patterns of structure and diversity in landraces of barley (*Hordeum vulgare L*) in a traditional agroecosystem in southern Ethiopia. The southern Ethiopian highlands present an ideal study system for this investigation, due to the region's mountainous and heterogeneous landscape, diversity of ethnic and cultural groups and social structures, high levels of crop diversity, and the continuing the presence of traditional seed exchange institutions such as local markets and gifting between family and neighbors.

### Crop population structure in traditional agricultural systems

Around the world, crop diversity is concentrated in a number of centers of origin and diversification, where evolution of crop species has occurred through millennia of interacting natural and human selection pressures, heterogeneous environments, isolation, migration, and farmer exchange (Harlan 1975). Farmers plant and replant seed, while selecting crops for yield, resistance to pests and disease, and interannual stability of production (Tsegaye 1997; Jarvis et al. 2011). The resulting farmer-identified crop varieties are genetically diverse and adaptable, allowing farmers to cope with heterogeneous environments and meet diverse production requirements and consumption needs (Bellon 1996). Genetic diversity of crop varieties has the potential to increase productivity, regulate nutrient cycling and microclimatic conditions, reduce temporal variability, and maintain resistance and resilience in the face of socioeconomic or environmental change (Altieri 1999; Shennan 2008).

The practices that farmers employ to access and save seeds, and the nature and location of seed sources through local markets and social networks determine dynamics of seed movement, mixing, and isolation that can create and maintain these diverse crops and varieties (Almekinders et al. 1994; Jarvis et al. 2011). Farmers exchange seed through a suite of methods and institutions, determined by social, economic, geographic, and environmental factors (Jensen et al. 2013), and these dynamics can determine the extent to which crop diversity is conserved on farms, and the potential for future *in situ* conservation (Thomas et al. 2011).

Many genetic analyses of landraces in farmers' fields find a lack of strong population structure; even when analyzing numerous communities across multiple regions, most diversity is located at the field level, underlay by larger clines of isolation-by-distance or directional geneflow (e.g., Pressoir and Berthaud 2004; van Etten et al. 2008; Pusadee et al. 2009). However, structure does exist, often corresponding with agroecological conditions, suggesting natural and farmer selection, or preferential gene flow within agroecological zones (vom Brocke et al. 2003; Parzies et al. 2004). Because, in these scenarios, gene flow is determined by farmer decisions, better understanding those decisions and their direct effects is a crucial aspect of conservation of crop genetic resources.

### Barley cultivation and diversity in Ethiopia

Barley is the 4th most cultivated cereal crop worldwide (Hubner et al. 2009). Much of this production is in the fields of small-scale farmers in marginal environments and developing nations. Barley is thought to have been domesticated in the fertile crescent region approximately 10 000 years ago and has been cultivated in Ethiopia for at least 5 000 years (Asfaw 2000). Ethiopia and Eritrea are considered a secondary site of diversification due to high levels of genetic and phenotypic diversity and strong genetic differentiation from Asian and north African populations (Orabi et al. 2007). The high morphological diversity of Ethiopian barley has been described by natural historians and scientists for nearly a century (Vavilov 1951). Farmers grow two- and six-rowed barley types in a range of characteristic colors and spike densities (Asfaw 2000). In addition to morphological diversity, researchers have identified high levels of diversity in biochemical composition (Demissie and Bjornstad 1996) and disease resistance (Negassa 1985).

Barley is a selfing species, although outcrossing rates vary within and among barley populations, ranging from <1% to more than 5%. Increased outcrossing rates have been observed, in Ethiopia and elsewhere, in situations of high abiotic stress or variable environmental conditions (Parzies et al. 2000; Abay et al. 2008). Despite high levels of selfing, farmer-maintained barley landraces are generally found to be highly variable, with the majority of genetic, morphological, and phenotypic variation found within fields and populations (Backes et al. 2009; Hadado et al. 2010).

In Ethiopia, barley is grown almost entirely by subsistence farmers and is cultivated on highland slopes up to 3500 meters above sea level (Lakew et al. 1997; Abay et al. 2011). More than 90% of the barley grown in the country is from farmers' landraces, rather than improved or breeder-produced varieties (Kebebew et al. 2001). Nationally, 95% of agricultural output comes from subsistence farms, and 69% of households farm on one hectare or less (CSA 2003).

### Research goals and questions

The goal of this study was to identify effects of farmers' seed exchange and management on the population structure of landrace barley in a traditional agricultural system in southern Ethiopia, using both farmer interviews and conservation genetic methods. We performed 121 farmer interviews on seed exchange patterns, and used these data to interpret the results of population genetic and phenotypic analysis.

We assess the extent to which barley population structure is correlated with two features of the system. First, we ask whether the region's steep elevation gradient affects the genetic structure of barley populations. Second, we ask whether the ways in which farmers manage barley seed are evident in barley population structure. Finally, we look at how farmer interviews can be used to shed light on genetic data, and ask whether this interdisciplinary methodology provides more information on patterns and processes of crop genetic diversity than the use of genetic methods alone.

## Methods

### Study Site

Part of the Gughe mountain range, the Gamo highlands rise out of the Ethiopian Rift Valley to elevations above 4000 m, in a chain roughly 100 km long (06˚ 02–27′N, 37˚10–37′E). Native vegetation includes mixed deciduous woodlands, dry evergreen montane forest, and alpine grasslands. Annual rainfall is bimodal, and mean annual temperatures range from 10°C to 25°C (MoA 2000).

These densely populated highlands are home to nearly one million people (FDRE 2008). The region is ethnically and linguistically homogeneous, representing the territory of the Gamo people and language. Within this ethnic group, there are clans, families, and traditional alliances that loosely, although not precisely, correspond to current political and management units. Two vehicle-accessible dirt roads cross the southern and northern reaches, while the central areas have little to no road access. The Gamo landscape, like much of Ethiopia, is dominated by small-scale subsistence agriculture. Household subsistence farms are organized into *kebeles* (called ‘communities’ here) of 500–1000 families. These are the smallest division of the federal government and are the scale at which many extension activities are organized. The Gamo highlands are spread across five districts, each made up of ten to twenty communities, within the Gamo-Gofa zone and the Southern Nations, Nationalities, and Peoples Region (SNNPR) of Ethiopia.

Agriculture in the Gamo highlands is based on a diverse combination of annual and perennial crops, agroforestry, and livestock management (Samberg et al. 2010). Barley is the most important cereal crop in the region, especially at high elevations where dozens of local varieties are identified by farmers for qualities of productivity, taste, texture, color, and row number. Farmers describe barley varieties in great detail, and take pride in their farms' diversity. At lower elevations, barley has less dietary and cultural significance and is grown in combination with wheat, maize, and sorghum. Barley management is necessarily viewed as one component in a larger system, and farmer decision-making takes place in light of numerous interacting management strategies.

### Farmer Interviews

**Figure 1:**
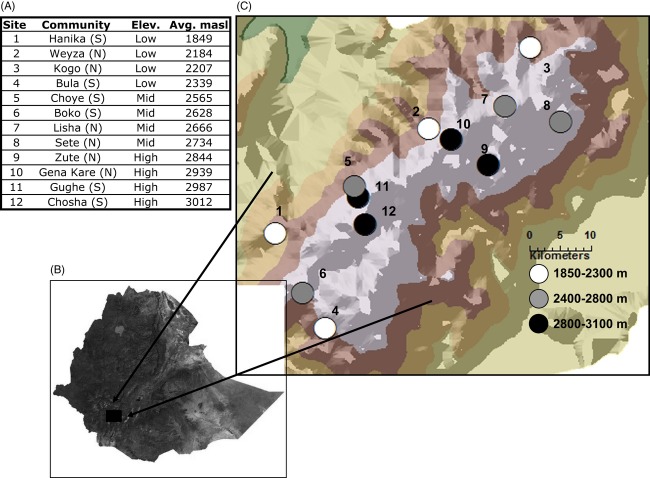
Map of study site: (A) sample communities, North/South location, and average elevation; (B) Gamo Highlands located in southwestern Ethiopia; (C) Twelve study communities located in the Gamo highland range, shaded by elevation class. Topographic shadings represent <1500, 1500–2000, 2000–2500, and 2500–3500 m ranges.

This research was carried out in twelve communities, representing five districts, in the Gamo highlands in 2008 and 2009. Communities were selected along the N-S transect of the highland plateau and are identified as ‘North’ or ‘South.’ Communities were also selected to represent three elevation classes, low (1800–2400 m), mid-altitude (2500–2700 m), and high (2800–3000 m) (Fig 1).

In 2008, 8–12 households were selected from each community, for a total of 121 farms. These households were selected represent a range of socioeconomic status and to include at least two female-headed households in each community. Semi-structured household interviews were administered by the research team on each sampled farm. Interviews were administered to the household head, along with any other adult household members who wished to participate. Interviews were extensive, and methods and results are reported in detail in a separate publication (Samberg et al. 2013). Farmers were asked to identify varieties present in each barley field, to identify the source of the planting material, the distance to that source, and the year of the most recent acquisition. They were also asked to explain their preferences for particular sources of seed, and their reasons for seed acquisition. Farmers were asked about their market habits, including number of markets visited, distance traveled to markets, and location of markets visited. In addition, the research team visited the eight largest markets in the region and identified barley varieties available for purchase, and the market price of each.

Interview data were analyzed qualitatively, for understanding of exchange patterns and rationale, as well as quantitatively, to determine the extent to which farmers engage in practices or hold opinions. Differences in farmer responses between sites, locations, elevation groups, and farm types were analyzed using anova and Student's *t*-tests using JMP statistical software (JMP, Version 9; SAS Inc., Cary, NC, 2008). The composition of barley varieties in each community was compared using a Bray–Curtis similarity index using EstimateS software (Colwell 2005).

### Barley collection and genotyping

In each of the twelve sample communities, barley fields were sampled in two consecutive years during the main barley growing season. In 2008, on each of the 121 surveyed farms, the research team visited all currently planted barley fields. Field sizes and the farmer's names for each variety were recorded. Leaf samples were then taken from two individual plants in each field, and preserved in CTAB solution for genetic analysis (Rogstad 1992). In 2009, the team returned to sample the three most common barley varieties in each community. For each of these, three fields were sampled, and five individual dried barley spikes were collected from each field. Samples are thus identified as being from one of 12 sites, located in the northern or southern portion of the range, and in one of the three elevation classes. In addition, they are identified as one of twelve farmer-named varieties. Varieties are identified in two major groups: eight varieties are six-row barley types, and four are two-row barley types.

Genomic DNA was extracted from leaf tissue samples from the 2008 field collection using a CTAB/chloroform protocol (Doyle and Doyle 1990) modified for use in 96-well format. Seeds from 540 plants collected in the 2009 season were grown in the glasshouse at the University of Montana, and tissue samples were collected from week-old seedlings for DNA extraction. Samples were analyzed at 14 microsatellite loci, two each on the seven chromosomes that make up the barley genome. Markers and primer sequences were drawn from Ramsay et al. (2000) and Jilal et al. (2008). Amplification was performed with amplification conditions suggested by Jilal et al. (2008). Genotyping was performed on an ABI Genetic Analyzer, and results were visualized and analyzed using ABI Genemapper software.

### Data analysis

Genetic diversity and structure of barley samples were assessed through both population-based and individual-based analysis. Genetic variation for sites, varieties, and elevation groups was assessed using measures of unbiased expected heterozygosity (*H*_e_). Genetic differentiation between sites, varieties, and elevation groups was estimated using *F*-statistics, and analysis of molecular variance (amova) was calculated to partition genetic diversity between sites and varieties. These analyses were performed using Genalex Software (Peakall and Smouse 2006). Linkage disequilibrium was calculated using Genepop software (Rousset and Raymond 1995).

Multiple linear regressions as well as Mantel tests were used to test the extent to which pairwise *F*_ST_ values and genetic distances between sites were correlated with geographic distances and differences in elevation. A principal components analysis (PCA) of allele frequencies at each community, variety, and variety within community was performed in JMP. Resulting axes were then analyzed for clustering by location and elevation.

Individual genotypes were then analyzed to identify cryptic population structures. Genalex was used to run a principal coordinate analysis (PCoA) on all multilocus genotypes, as well as on samples from six-rowed and two-rowed varieties separately. Significant differences in values for PCoA axes were used to identify distinct population segments.

A Bayesian analysis of population structure, without *a priori* population identification, was carried out in STRUCTURE software (Pritchard et al. 2000), in order to confirm the nature of subpopulations identified in *F*_ST_ analysis and PCoA. Although STRUCTURE assumes Hardy–Weinberg equilibrium, it has been used repeatedly to analyze barley and other selfing species that violate this assumption (e.g., Backes et al. 2009; Hubner et al. 2009). Gao et al. (2007) have considered this problem and conclude that STRUCTURE, when used with caution, can provide meaningful analysis with selfing species. STRUCTURE analyses were run on the entire sample set, as well as for six- and two-rowed varieties separately. Analyses were run with a burn-in of 50 000 rounds and an additional 50 000 repeats at ten iterations for each value of K = 2–10. Likely values of K were identified through analysis of the rate of change in the likelihood of each value of K (as per Evanno et al. 2005) as well as the relevance of assignments to meaningful geographic, elevation, or variety patterns.

### Phenotypic analysis

Seed from the 540 individual plants collected in the 2009 field season was planted in a common field at Montana State University in Bozeman, MT in the summer of 2010. Each plant head was assigned a random identification number, threshed by hand, and all seed from each individual head was planted in randomized plots in two rows of a larger barley field. The progeny of each individual were assessed for several quantitative and qualitative traits: days to heading, plant height, 1000-seed weight, row number, and seed color. Differences in plant height, days to heading, and seed weight between sites, elevation groups, and varieties were tested for significance using full-factorial anova as well as individual anova and t-tests for elevation classes and North/South location.

## Results

### Farmer-identified barley diversity and seed exchange practices

Farmers identified 33 named barley varieties, split between six-rowed types grown in larger fields for household consumption, exchange between famers, and occasional sale at market, and two-rowed types grown in small plots for specific cultural or medicinal uses. The average number of barley varieties on-farm is 2.3, and on-farm diversity of barley varieties increases strongly with elevation, with an average of 1.7 varieties on low-elevation farms, and 2.8 varieties on high-elevation farms (Table 1).

**Table 1:** Interview data on barley diversity, field size, and seed exchange practices, compared by location and elevation group

	Total	North	South	Low	Mid	High
Avg # of barley varieties per farm	2.27	2.28	2.26	**1.43**	2.55	2.77
Avg size of barley fields (m^2^)	1010	791	1205	959	824	1341
Avg size of 6-row barley fields (m^2^)	1297	**948**	**1631**	1290	1307	1598
Avg size of 2-row barley fields (m^2^)	436	**528**	**212**	409	341	570
% of farmers responding						
Farmers acquired barley seed in past year	42	40	43	**53**	37	36
Farmers getting new seed due to loss	69	66	73	79	69	59
Farmers getting new seed for experimentation	24	24	24	**14**	31	31
% of barley fields surveyed						
Planted from own seed	55	**62**	**49**	**39**	62	58
Planted from Neighbors' seed	16	13	18	23	14	14
Planted from market seed	25	**19**	**30**	**36**	24	21

Quantities represent averages over the group (# of barley varieties and field size), percent of respondents in that group, or percent of barley fields surveyed in that group. Bold figures represent significant differences from other locations or elevations at α = .05.

Over the course of this survey, 303 barley seed lots currently planted in the field were investigated. The average size of fields for six-rowed varieties was 1297 m^2^; while the average size of fields for two-rowed varieties was 436 m^2^ (Table 1). Of all fields, 55% were reported to be replanted with the farmer's own seed from the previous year. This number increased with elevation, with higher elevation farms reporting greater levels of seed security (Table 1).

One quarter of barley fields were planted with seed from local markets, and this percentage decreases at higher elevations. Households in the Gamo attend, on average, four different markets each week, and communities share markets with nearby communities. Six-rowed varieties were more widespread in the markets, with each variety found in an average of 4.8 markets of the eight surveyed markets, while two-rowed varieties were found in an average of two markets each. Six-rowed varieties were also less expensive, sold for an average of 2.7 Ethiopian birr per kg, while seed for two-rowed varieties sold for an average of 4.2 birr per kg. The distribution of named barley varieties across the Gamo landscape, as measured by a Bray–Curtis index of similarity between variety names in each community (Bray and Curtis 1957), is tightly correlated with elevation differences between communities (*R*^*2*^* *=* *0.25, *P *<* *0.001), as well as with geographic distance (*R*^*2*^* *= 0.10, *P *=* *0.007). According to surveyed farmers, seed flow largely occurs between communities of the same elevation, and farmers identify distinctive low- and high-elevation varieties.

### Genetic variation within populations

Samples were genotyped at 14 microsatellite loci, with a total of 132 alleles (Table 2). The most polymorphic locus (*Bmac223*) had 25 alleles; the least (*Bmac579* and *Bmac316*) had four each. One locus (*Bmac136*) did not amplify in the majority of samples and is excluded from further analyses.

**Table 2:** Microsatellite markers used in this study, the chromosome on which they are located, the number of alleles found at that locus across all samples, and *F*_st_ for site and variety group for each locus

Marker	Chr.	# of alleles	*F*_st_ (Sites)	*F*_st_ (Varieties)
Bmac0032	1	13	0.131	0.173
Bmag0579	1	4	0.361	0.578
Bmac0134	2	11	0.068	0.119
Bmac0093	2	7	0.136	0.245
Bmac0067	3	5	0.129	0.286
Bmag136	3	8 + null	–	–
Bmac310	4	5	0.143	0.286
Bmag0353	4	8	0.148	0.369
Bmac0096	5	8	0.126	0.299
Bmag0223	5	26	0.083	0.151
Bmac0316	6	9	0.089	0.182
Bmag173	6	8	0.088	0.154
Bmag0206	7	17	0.112	0.149
Bmag120	7	11	0.109	0.201

Approximately 1 in 20 samples (4.7%) was heterozygous at one or more loci, reflecting low levels of outcrossing. *F*_IS_ values ranged from 0.95 to 1 for sites and loci (Table 3). Despite low levels of observed heterozygosity, all individual fields contained a mixture of multilocus genotypes, and 68% of genotypes were unique among the samples. Significant linkage disequilibrium was found between all pairs of loci, with loci in the same linkage group no more tightly linked than any other pair of loci.

**Table 3:** Expected Heterozygosity, *F*_is_, and *F*_st_ for sites, elevations, and locations, and farmer-named varieties

Site	Elevation	N/S	H_e_	*F*_is_	*F*_st_
Hanika	Low	S	0.54	0.99	**0.15**
Weyza	Low	N	0.63	0.99	0.06
Kogo	Low	N	0.60	0.99	0.06
Bula	Low	S	0.49	0.98	**0.10**
Choye	Mid	S	0.63	0.99	0.04
Boko	Mid	S	0.52	0.99	0.05
Lisha	Mid	N	0.51	1.00	0.07
Sete	Mid	N	0.52	0.98	0.06
Zute	High	N	0.55	0.98	0.05
Gena Kare	High	N	0.49	1.00	0.06
Gughe	High	S	0.56	0.99	0.06
Chosha	High	S	0.51	1.00	0.05
Low	Low	All	0.65	0.99	**0.10**
Mid	Mid	All	0.57	0.99	0.03
High	High	All	0.55	0.99	0.04
North	All	N	0.59	0.99	0.02
South	All	S	0.61	0.99	0.02
Variety		Row #			
Shilasho	Low	Mixed	0.61	0.99	**0.12**
Ufale	Low	Mixed	0.67	0.99	0.09
Wariwacho	Low	6	0.48	0.99	**0.19**
Bote	All	6	0.54	0.99	0.07
Chega	All	6	0.57	0.99	0.06
Gajeta	High	6	0.49	0.99	0.07
Kazha	High	6	0.55	0.97	**0.10**
Wolate	High	6	0.56	0.99	0.07
Solga	Low & Mid	2	0.50	0.99	**0.10**
Koltso	Mid	2	0.42	0.97	**0.12**
Ocho	Mid & High	2	0.48	0.99	**0.10**
Wake	Mid & High	2	0.40	0.99	**0.13**

Bold *F*_st_ values represent significant population differentiation at α = 0.05.

Expected heterozygosity (*H*_e_) at each site ranged from 0.49 to 0.63 (Table 3). Low-elevation sites had higher expected heterozygosity (*H*_e_ = 0.65) than mid- and high-elevation sites (*H*_e_ = 0.57 and 0.55). The lowest levels of heterozygosity were found in two two-rowed varieties (*H*_e_ = 0.40 and 0.42), while the highest were found in varieties of mixed row type found only at low elevations (*H*_e_ = 0.61 and 0.67).

### Population genetic structure

Analysis of molecular variance (amova) partitions 11% of diversity between sites, indicating low but significant structure at the community scale. Overall *F*_ST_ between communities at all loci is 0.137, and pairwise *F*_ST_ values ranged from 0.02 to 0.15. *F*_ST_ values at each locus range from 0.08 to 0.15, with one outlier (*Bmac579*) at 0.35.

Structure between farmer-named varieties represents 15% of the genetic variance, based on amova, and *F*_ST_ between farmer-named varieties is 0.246, indicating structure by variety name. *F*_ST_ at each locus ranged from 0.15 to 0.37, with the exception of *Bmac 579*, again an outlier with an *F*_ST_ of 0.58 across all varieties. Pairwise *F*_ST_ values between sites, used as a measure of genetic distance, increase significantly with the difference in elevation between sites (*P* < 0.01), and to a lesser extent with geographic distance between sites (*P* < 0.03) based on Mantel tests.

**Figure 2:**
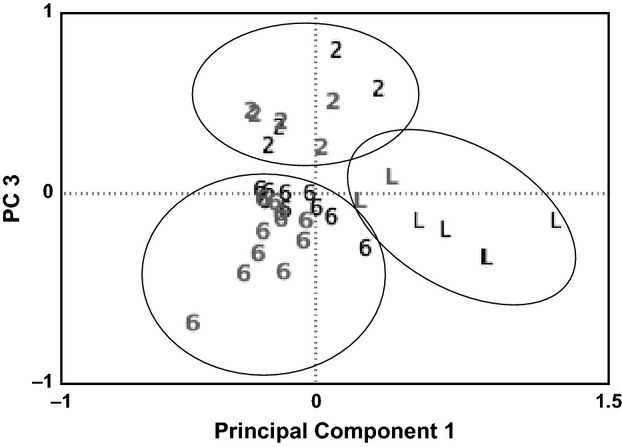
First and third PCA axes for covariance of allele frequencies for each site and variety show clustering by 6-row (6), 2-row (2), and distinctly low populations (L). Northern (Gray) and southern (Black) sites are identified.

Principal component analysis (PCA) of allele frequencies of sites and varieties identified three distinct clusters (Fig. 2). The first axis, accounting for 17% of variation, distinguished distinctly low-elevation varieties, which are mixed two- and six-row varieties found only in low-elevation communities, while common varieties found at low elevations are not distinct. The third axis, accounting for 11% of variation, differentiated six-rowed and two-rowed barley types. These three groups display distinct spatial patterns and are discussed in turn.

### Low-elevation varieties

**Figure 3:**
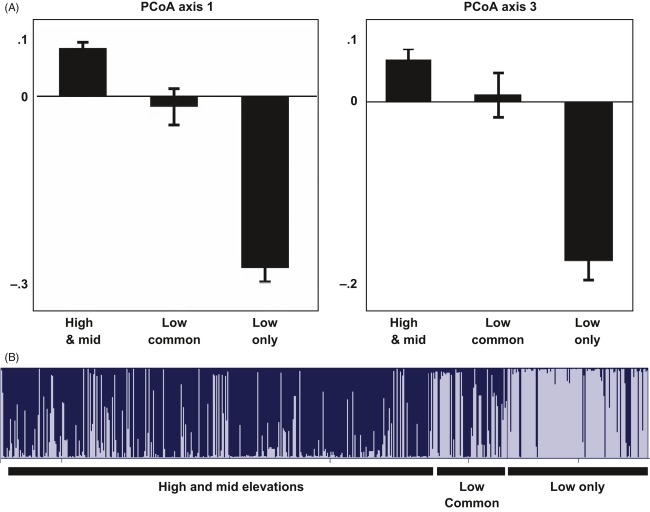
Distinctly low populations are differentiated from both high- and mid-elevation samples and samples of common varieties found in low sites. (A) Low varieties show differentiation on the first and third axes of a PCoA on all individual genotypes. Mean and standard error of values for each group are shown. (B) STRUCTURE analysis identifies the majority of low-variety samples as belonging to a distinct population; common low samples are split between the two inferred populations.

The most evident pattern in both population-based and individual-based analyses is the distinctiveness of barley varieties unique to low populations. A principal coordinate analysis (PCoA) of 682 multilocus genotypes displays the distinct nature of low-elevation varieties (Fig. 3A). Genotypes were analyzed using STRUCTURE software to test the existence of this more cryptic structure. Analysis of δk identified k = 2 as the most likely structure, with 85% of samples from low-elevation varieties assigned to a distinct population, as compared with 38% of samples from common varieties from low elevations, 16% of six-rowed varieties from high and mid-elevations, and 4% of two-rowed varieties (Fig. 3B). In addition, low-elevation populations from the southern portion of the range were more distinct than those from the north, as seen in the PCA (Fig. 2), as well as the individual PCoA on genotypes.

### Six-rowed varieties display large-scale spatial structure

With the removal of specific low-elevation varieties, the remaining six-rowed barley varieties across eleven sites show little spatial structure based on *a priori* populations. One low-elevation site (Bula) remains as an *F*_ST_ outlier; without this site, pairwise *F*_ST_ values between sites are not significantly correlated with elevation differences, and *F*_ST_ among the remaining sites is 0.09. amova indicates that sites account for 8% of variation, while variety names account for 5% of variation.

However, pairwise *F*_ST_ values between communities retain a weak but significant relationship with distance (*P* < 0.01), and PCA of allele frequencies for sites and varieties show both north–south and elevation patterns in these varieties. Individual PCoA of genotypes also identifies weak, but significant clustering along north–south and elevation gradients (*P* < 0.001).

**Figure 4:**
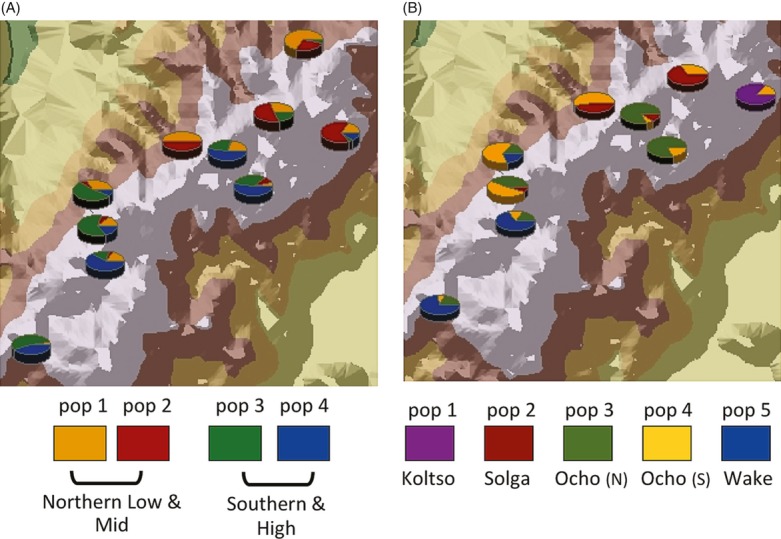
Proportion of samples at each site assigned to inferred populations in STRUCTURE. (A) 6-rowed varieties: the four geographically meaningful populations show a gradient of membership. The fifth population is not shown. (B) 2-rowed varieties: Membership in each of the five inferred populations indicates clustering between nearby sites.

STRUCTURE analysis at k = 2 through 10 do not indicate population assignments associated with site or variety. Using the δk method, the most likely structure lies at k = 5, and analysis identifies a clinal pattern across the region, as inferred populations are drawn disproportionately from northern or southern sites, or high or mid-elevations. Two inferred populations contain nearly entirely northern low- and mid-elevation individuals, while two contain a mix of high-elevation northern samples and southern samples from all elevations, indicating a gradient across the region (Fig. 4A). The fifth population shows no clear pattern. These results support the identification of structure at a scale larger than that of the community or variety.

### Two-rowed varieties show structure by distance and variety name

**Figure 5:**
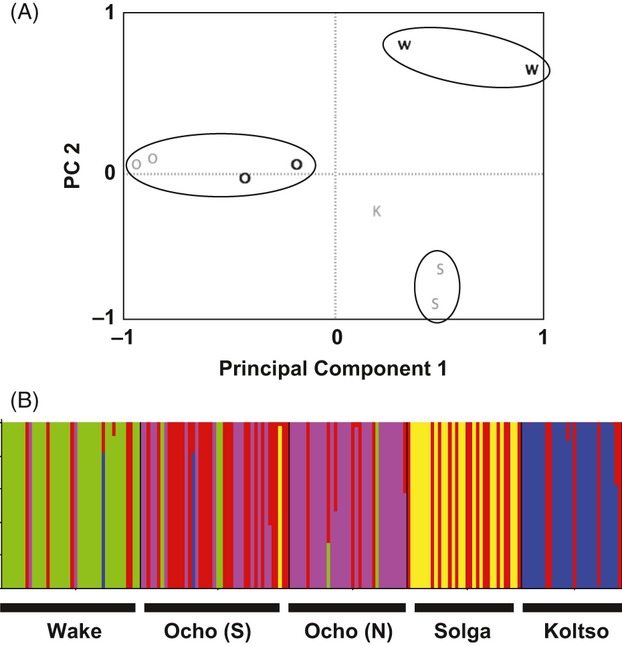
Two-row varieties are differentiated by farmer names. (A) First and second PCA axes on covariance of allele frequencies of 2-rowed samples by site and variety. Populations are labeled as one of four 2-rowed varieties: Ocho (O), Wake (W), Solga (S), and Koltso (K), and cluster by variety name. Populations identified by north (light) and south (dark). (B) STRUCTURE analysis of 2-rowed varieties, at k = 5, shows inferred populations corresponding to variety names. The Ocho variety, the most widespread, appears as a distinct population in the northern portion of the range, and as a mixture in the southern portion of the range.

The four-two-rowed varieties in our study were located across nine communities. The overall *F*_ST_ between communities was 0.222, and pairwise *F*_ST_ values ranged from 0.039 to 0.224. amova partitions 23% of variation between communities, indicating strong differentiation. Pairwise *F*_ST_ was correlated with geographic distance between communities (R^2^ = 0.25; *P* = 0.002). In addition, contrary to findings in six-rowed samples, variety names were correlated with genetic differentiation, with amova partitioning 25% of genetic variation between varieties. PCA of allele frequencies for each variety and community (Fig. 5A) and PCoA of individual genotypes display distinct groupings by variety name.

Analysis in STRUCTURE also identified populations based on farmer-named varieties. The most likely assignment occurs at k = 5, with four distinct populations coinciding with named varieties (Fig. 5B). 80% of the *Wake* variety is assigned to population 1, 60% of samples from the *Solga* variety are assigned to population 3, and 80% of the *Koltso* variety is assigned to population 4. For the most common variety, *Ocho*, 80% of samples from the north are assigned to population 2, while samples from the south are split between that population and the fifth inferred population, which is made up of individuals from each of the four varieties, especially from the northern variety, *Solga*. Spatially, these inferred populations indicate discrete clustering of nearby communities or single communities (Fig. 4B).

### Phenotypic patterns

The progeny of the 540 barley samples grown in the field at the research station were analyzed for the characteristics used by farmers for identification. Heads harvested from each sample were assessed for row number and seed color. Farmer variety names most often described a consistent row number and seed color, with the exception of two distinct low-elevation varieties, *Shilasho* and *Ufale*, which were generally identified as six-rowed types, but in some communities described two-rowed types.

Plant heights were distributed normally between 56 and 98 cm. While there were significant differences between varieties, with two-row types significantly taller, there was no clear trend of site or location. Two-rowed types originating at high elevations were, on average, shorter, creating the only elevation-based pattern evident in plant heights (Table 4).

**Table 4:** Average plant height, days to heading, and 1000-seed weight for 2- and 6-rowed varieties; overall, and by location and elevation

	Plant height (cm)		Days to Heading		1000 seed weight (g)	
	6 row	2 row	6 row	2 row	6 row	2 row
Total	75.9	80.4	60.5	61.1	45.4	57.7
North	75.2	81.3	59.7*	61	47.3*	57.7
South	76.6	79.4	61.4*	61.2	43.7*	57.6
High	74.3	78.2*	62.1*	62.4*	45.2	59.3
Mid	74.5	82.4	59.5	60.3	46.4*	59.9
Low	75.2	81.5	60.1	60.3	44.8	52.3*

* Significant differences between locations or elevation classes.

Plants took between 56 and 66 days from planting to heading. Plants from communities at high elevations, both six- and two-rowed, took significantly longer to head than plants from mid or low elevations (Table 4). The uniquely low varieties had significantly lower seed weights than those at mid and high elevations, both for two- and six-rowed types, although common varieties found at low elevations did not weigh significantly less than their higher-elevation counterparts.

**Figure 6:**
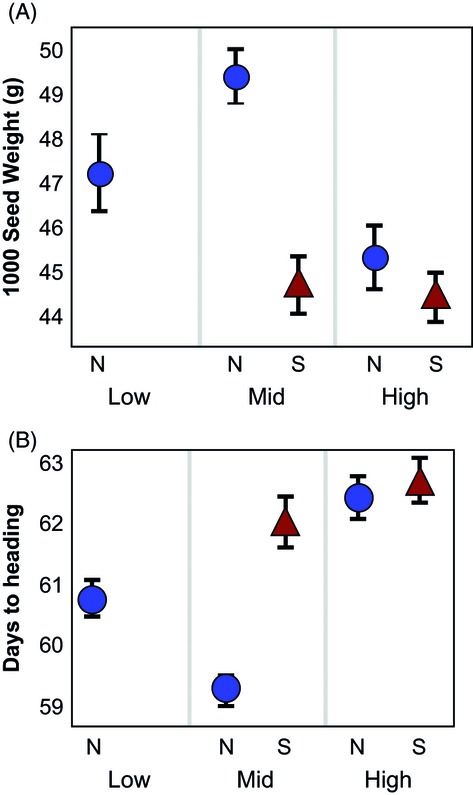
Six-row varieties show differentiation in phenotypic traits between low- and mid-elevation northern sites, and high elevation and southern sites. Northern sites indicated with circles; southern sites with triangles. Mid- and low northern sites have (A) higher seed weights and (B) shorter flowering times. Points indicate mean values ± 1 SE.

For common six-rowed varieties, seed weight and flowering time traits showed geographic patterns coincident with the observed genetic structure. Samples from northern mid- and low elevations had higher seed weights and shorter flower times than samples from high elevations and southern sites, the same split identified by STRUCTURE (Fig. 6). Among two-rowed types, named varieties differed significantly in weight (*P *<* *0.01); however, there was no differentiation by location or elevation.

## Discussion

Our results indicate disparate patterns of exchange and isolation between elevation zones and barley types, with two patterns arising. The first is the distinctiveness and diversity of low-elevation varieties. The second is the marked difference in population structure between six- and two-rowed barley types, which indicates that farmer management affects crop population structure, independent of the physical landscape.

### Structure by elevation

Samples from low-elevation communities differ from those at high- and mid-elevation communities at several loci, and the two southern low communities especially have significantly different allele frequencies from other sites and from each other. PCoA and STRUCTURE analysis show that differentiation of low-elevation communities is driven by a specific set of varieties that are grown only at these elevations, while more widespread varieties do not show structure by elevation. This indicates that seed exchange does occur across altitudinal gradients for some named varieties. Population structure corresponding to elevation is common in landrace barley. In India, altitude was seen to be the largest factor in the structure of landrace barley populations (Pandey et al. 2006), and a study of Spanish barley identified temperature (tightly correlated with elevation) to be the main factor affecting the distribution of alleles (Yahiaoui et al. 2008). In Ethiopia, a recent study found that differentiation among altitude classes was several times higher than differentiation among geographic districts, with the greatest amount of population differentiation found in the lowest altitude class (Hadado et al. 2010). In addition, several studies of Ethiopian barley have shown strong association of phenotypic traits with altitude (Alemayehu and Parlevliet 1997; Kebebew et al. 2001).

The similarity of low-elevation varieties throughout the study area at several loci, and the relative similarity of high-elevation communities to one another, also suggests the possibility that there is differential selection occurring between high and low communities. Recent studies of crop landraces have shown patterns of asymmetrical adaptation, in which high altitude populations are more narrowly adapted than low-elevation populations (Mercer et al. 2008). A recent study of barley landraces in northern Ethiopia also suggests a signal of homogenizing local adaptation at high elevations (Hadado et al. 2010).

In this study, high-elevation samples of all types have longer flowering times than their lower-elevation counterparts (Table 4, Fig. 6). As growing seasons are longer at high elevations in the Gamo, this indicates potential local adaptation in these samples. Barley is a crucial part of household subsistence at high elevations, and strong farmer selection to maximize productivity in each location could drive this divergent pressure.

Farmers in low-elevation communities may select for a different set of conditions, or may not employ strong selection, allowing diverse genotypes to remain in the population. The diversity of both alleles and phenotypic characteristics found within communities and named varieties, and the fact that low-elevation varieties have lower seed weight, indicating potentially lower grain quality, suggest a lack of strong directional selection at low elevations.

### Structure based on farmer management

Six- and two-rowed barley populations have distinctly different population structures, suggesting that differential management and exchange strategies can lead to different spatial genetic patterns on the same physical landscape.

Two-rowed varieties show spatial clustering between nearby communities, indicating movement between neighboring communities, although not over long distances, and a lack of strong barriers to exchange due to elevation. Two-rowed barley also shows distinct clustering by farmer-named variety. PCA and STRUCTURE analysis indicate that variety names describe genetically distinguishable entities, even in cases in which physical characteristics such as seed color and size are similar between varieties, implying attention to variety management in these types. The phenotypic data also distinguish between named varieties, most clearly in seed weight.

Six-rowed varieties, however, show a much larger-scale spatial pattern. Neither sites nor varieties represent distinct genetic entities. Instead, there is a broader pattern spanning the northern and southern portions of the range, as well as the elevation gradient. These patterns are evident both in the genetic data and the phenotypic data, with significant differences between northern low and mid-elevation populations and southern and high populations (Figs 5A and 6). Both the weakness and the geographic scale of this pattern indicate high levels of seed exchange and geneflow across the range of the Gamo highlands in six-rowed varieties. The greater presence of six-rowed varieties in local markets, and the lower price of seed for these varieties, supports the existence of greater levels of exchange over distance.

That variety names do not play a meaningful role in the genetic structure of these varieties indicate that there is significant gene flow between them, despite identifiable differences in seed color and other characteristics used by farmers for identification. However, it is quite plausible that landraces may retain distinct morphological characteristics in the face of high geneflow, even when neutral variation does not distinguish between them. Farmers select seed based on specific identifiable traits for each variety, and therefore, those varieties may share the specific genes responsible for this distinctiveness, while showing little differentiation at neutral loci (Barnaud et al. 2007).

### Importance of interview data

There are several features of barley seed exchange, as discussed by farmers throughout the interview process, that provide insight into the observed patterns in barley population structure. The first is farmer focus on perceived agroecological adaptation when acquiring seed. The elevation from which seed originates is the primary factor used by farmers in seed acquisition, and thus, seed exchange occurs more frequently and in greater volumes between farmers and communities at similar elevations. This practice is likely related to the genetic isolation of barley in low-elevation communities.

Interviews indicated that, as barley is less important at low elevations, not only do low-elevation farms cultivate fewer barley varieties, they are also less seed secure for barley. At high elevations, lower levels of seed turnover create conditions for greater selection, both natural and by farmers, and therefore greater adaptation to local conditions. These practices, and the lesser importance of barley to low-elevation farmers in the Gamo, may shed light on the different differences in diversity and structure of high- and low-elevation barley.

The second lesson learned through the interview process is the differentiation in strategies used by farmers in the management of two-rowed and six-rowed types. The differential use and management strategies large plots, often intermixed with new seed, and selected for productivity, as opposed to small plots cultivated for specific taste, color, and texture qualities may be responsible for the difference in population structure between these barley types. The greater prevalence and lower price of six-rowed varieties in markets suggests that markets are a stronger force in the structuring of six-rowed varieties, and the long-distance exchange evident in genetic and phenotypic patterns. Farmers are less dependent on markets for the procurement of seed for two-rowed varieties, acquiring seed from friends, family, and neighbors. This strategy provides a level of explanation for both the tight clustering between nearby communities and the genetic fidelity to variety names found in two-rowed varieties.

These farmer interviews provide explanatory information for the observed genetic patterns, without which the genetic data may be misinterpreted or cryptic. The structure seen in these populations cannot be understood purely in the context of physical barriers or divergent selection pressures. Interpretation requires knowledge of reluctance to exchange seed with low-elevation communities, and differential management for two- and six-rowed varieties, which can only be gleaned through concurrent social science methods.

## Conclusions

Landscape and conservation genetic methods are increasingly being adapted for use in human-influenced systems, such as those where agriculture, livestock husbandry and grazing, hunting, and harvesting are key components. In these landscapes, neither the physical landscape nor the biology of the organism of interest can fully explain the patterns and processes of population structure; rather, the social landscape of farmer management and exchange determines the distribution of genetic diversity of numerous species. In addition, conservation scientists and practitioners in agricultural landscapes are increasingly recognizing the importance of considering farmers' economic and cultural needs in designing appropriate strategies (Garcia et al. 2009).

In the Gamo highlands, farmer management of seed and seed exchange leads to three distinct sets of population processes in the same physical landscape: isolation and diversification of low-elevation varieties, broad geographic patterns in productive varieties, and clustering by location and variety name in specialty varieties. Farmer interviews regarding management provide crucial information about the extent and directionality of seed flow and factors influencing selection. These social landscape features interact with steep altitudinal gradients over space to create specific genetic patterns, and those patterns and processes will differ between landscapes, species, and societies. An understanding of the social landscape is required to both capture the underlying ecology and to design appropriate strategies for conservation.

## Data archiving statement

Raw data for this manuscript are available in Dryad: doi: 10.5061/dryad.nk143.
